# Robotic Surgery may Not “Make the Cut” in Pediatrics

**DOI:** 10.3389/fped.2015.00010

**Published:** 2015-02-12

**Authors:** Nicholas E. Bruns, Oliver S. Soldes, Todd A. Ponsky

**Affiliations:** ^1^Department of Pediatric Surgery, Akron Children’s Hospital, Akron, OH, USA

**Keywords:** robotic surgery, robot, pediatric, children, minimally invasive surgery, laparoscopy, thoracoscopy

## Abstract

Since the introduction of robotic surgery in children in 2001, it has been employed by select pediatric laparoscopic surgeons, but not to the degree of adult surgical specialists. It has been suggested that the technical capabilities of the robot may be ideal for complex pediatric surgical cases that require intricate dissection. However, due to the size constraints of the robot for small pediatric patients, the tight financial margins that pediatric hospitals face, and the lack of high level data displaying patient benefit when compared to conventional laparoscopic surgery, it may be some time before the robotic surgical platform is widely embraced in pediatric surgical practice.

## Introduction

Robotic minimally invasive surgery has been rapidly adopted for a wide variety of surgical procedures in adult patients across a broad spectrum of surgical specialties. This has occurred despite the high costs and uncertain benefits of surgical robots (1). Local competitive pressures may be driving the purchase of a robot. Adoption of this technology by a neighboring hospital increases the likelihood of nearby hospitals acquiring a surgical robot (1). In contrast, Children’s Hospitals and pediatric surgical disciplines have been much slower to embrace the surgical robot. Many children’s hospitals do not even possess a surgical robot, and many of those that do borrow them from the adult operating room within the same medical facility (2). This review examines the history of surgical robots in pediatric surgery, the potential benefits, and the technical, financial, and other barriers to adoption of this technology.

## History

The first case of robotic minimally invasive surgery in children was a Nissen fundoplication that was performed in July 2000 and reported in April 2001 (3). Since that time, robotic procedures have been slowly adopted by select pediatric surgical specialists. In the following decade, there were a total of 2393 procedures reported in 1840 patients in the published literature (4). The most common gastrointestinal and thoracic procedures were fundoplication (424) and lobectomy (18), respectively (4). Pyeloplasty was the most common procedure overall (672) (4). Of the 137 reviewed publications, 122 (89%) utilized the da Vinci Surgical System (Intuitive Surgical, Inc.), making it by far the most prevalent and most studied robotic platform (4). In comparison, there were over 400,000 procedures performed in adults on the da Vinci system in the last year alone (5). Thus, adoption of robotic surgery is decidedly less common in the pediatric surgical specialties relative to the adult surgical disciplines.

## Technical Benefits

Advocates of robotic minimally invasive surgical systems tout many useful features that include improved dexterity, motion scaling, tremor filtration, greater optical magnification (up to 10×), stereoscopic vision, operator-controlled camera movement, and the elimination of the fulcrum effect when compared to conventional laparoscopy (6–8). The wristed laparoscopic instruments used in robotic surgery provide seven degrees of freedom. For the surgeon, these features may allow for more precise dissection with increased magnification and visibility. The intuitive controls of the robot are purported as providing the ability to perform laparoscopic procedures in an “open” fashion. In pediatric surgical procedures, these technical abilities may have the potential to surpass the physical capabilities of human performance in the tight operative fields encountered in children.

## Technical Limitations

Many of the most challenging and complex procedures, where robotic minimally invasive surgery may hold the most potential, are performed in newborns. It seems intuitive that the surgical robot would be optimal for the small operative spaces in pediatrics. However, its technical requirements can make it cumbersome or not feasible for smaller patients. The manufacturer of the da Vinci surgical robot recommends an 8 cm distance between each port. This may be difficult to achieve in many neonatal cases. The size and length of the instruments can be an issue as well. Neonatal surgical procedures are often performed with 3-mm instruments and endoscopes, which are smaller than the smallest instruments and endoscopes available currently for robotic surgery.

Currently, there are two endoscopes available for the da Vinci Surgical System: 12-mm 3D and 8.5-mm 3D scope. There was also a 5-mm 2D scope that was developed and later discontinued due to low usage. The 8.5-mm scope may be more versatile for smaller children, but it is still large for the intercostal space of a 5 kg child (9). Instruments are available in two sizes: 8 and 5 mm. The 8-mm instruments articulate with a pitch-roll-yaw mechanism, whereas the 5-mm instruments articulate in a snake-like manner (10). The difference in articulation results in the 5-mm instruments being longer than their 8-mm counterparts, losing workspace within a small body cavity. For infants and toddlers, 3-mm instruments are routinely used for many basic and advanced laparoscopic procedures. The lack of commercially available 3-mm instruments is a significant limitation of the current robotic surgical platforms and a disincentive for their use in small children. Finally, there are a limited number of instruments from which to choose. According to the instrument catalog from January 2013, there are forty 8-mm instruments and twelve 5-mm instruments. For many pediatric surgeons, creating the smallest possible incision is a major advantage of laparoscopic procedures. The absence of 3-mm and the few options for 5-mm instruments may limit the use of the robots in infants and toddlers.

## Patient Benefits

The patient benefits of robotic surgery are thought to be essentially the same as conventional laparoscopy: decreased length of stay, decreased blood loss, decreased pain, quicker return to work, and improved cosmetic result through smaller incisions (11). In pediatric urology, there is evidence that robot-assisted pyeloplasty may be superior to open and laparoscopic approach with decreased length of stay, decreased narcotic use, and decreased operative times (12, 13). In an analysis of the Nationwide Inpatient Sample that compared robotic to laparoscopic and open surgery, among most procedure types, there was a significant decrease in the length of stay and likelihood of mortality for the robotic surgery group when compared to the open and conventional laparoscopic surgery groups (14). However, the effect was significantly diminished when comparing robotic to laparoscopic surgery alone. In the robotic surgery group, the length of stay was 0.6 days shorter with increased charges of $1300 (14). A recent randomized controlled trial in adults comparing open to robotic surgery showed no difference between the groups in terms of complication rate and length hospital stay for radical prostatectomy (15). There was a significant difference in operative time: the open group was 2 hours shorter (15). These findings suggest some potential benefits to robotic surgery, but additional study is needed to verify the benefits, especially with respect to laparoscopic surgery. There are no randomized controlled trials in the pediatric population.

## Costs

Robotic surgery has higher costs than open and laparoscopic procedures. This is due to the high costs of purchasing and maintaining a robot, increased operative time, and costs of disposable surgical supplies (16). In a retrospective analysis of 368,239 patients from the Nationwide Inpatient Sample database, there was an increase in total charges of $1309 per robotic-assisted case (14). The da Vinci Surgical Systems typically cost between $1.0 and $2.3 million and require an additional maintenance contract of $100,000 to $170,000 per year in addition to variable disposable instrument costs (5). In the US medical system of reimbursement, these extra costs may result in robotic procedures being financially unfeasible given the slim operating margins (<1%) on patient care of most US hospitals. This has been described in a single institution cost analysis of robotic radical prostatectomies that showed a net loss of $4013 for the robotic procedure compared to a net profit of $1325 for the open procedure (17). According to 2012 AHA Annual Survey, the aggregate operating margin of all hospitals was 6.5% and the patient care margin was only 0.7% (18). For pediatric hospitals, these margins may be even slimmer. For example, at Akron Children’s Hospital, 49.5% of all revenue was paid by Medicaid, which is known to have lower reimbursement rates (19). Given the modest margins on patient care, hospitals strive to minimize costs. For surgical procedures, this may be accomplished by decreasing operative time or length of stay (16).

At a stand-alone pediatric hospital, a robotic platform is often not available. Although Intuitive Surgical does not release exact sales figures for pediatric hospitals, it is safe to assume only a minority have robotic systems given the limited number of procedures performed nationally. This is probably due to the costs of acquiring and maintaining a surgical robot coupled with the tendency for pediatric hospitals to have less income and fewer eligible patients to defray the fixed costs of the platform. A unique situation exists for pediatric surgeons in hospitals affiliated with adult care as robots may be available that are primarily used for adult subspecialties, most often urology (2). In this setup, the logistics may be difficult and the pediatric team must be flexible and mobile to accommodate the robot (2).

## Safety

The overall reported conversion-to-open-procedure rate is low. It has been reported as 2.5% in a meta-analysis of robotic pediatric surgery (4). When broken into subgroups, it was 3.9, 1.3, and 10% in gastrointestinal, genitourinary, and thoracic cases, respectively (4). This is comparable to the conversion rate in conventional pediatric minimally invasive surgery (20). However, the real conversion rate for robotic pediatric surgery may be higher due to citation bias. As well, there has been evidence of underreporting of complications following robotic surgery in both the media and medical literature. Over a 12-year period, a review that cross-referenced device-related complication databases with the FDA revealed 8 cases that were either unreported or incorrectly reported (21). This is especially concerning because the true incidence of device-related complication is unknown.

## Learning Curve

Laparoscopy has been adopted for advantages that include decreased adhesion formation, improved cosmesis, decreased post-operative pain, and shorter recovery times (11). A skilled laparoscopic surgeon may see no additional benefit when compared to robotic surgery (8, 22). In a study comparing novice and expert surgeons that completed tasks on laparoscopic and robotic simulators, there was a significant improvement in speed and smoothness of performance in the novice group when using the robot that was not replicated in the expert group (22). This suggests that the novice laparoscopist may realize the greatest benefit of robotic surgery. There is evidence that a learning curve is encountered when adopting robotic surgery as demonstrated by decreasing operative times as case volumes increased (2). This learning curve may be more difficult to surpass for the most complex neonatal congenital surgical cases such as tracheoesophageal fistula repair where even the busiest pediatric surgeons do only a handful of such cases per year due to the low incidence of the condition (23).

## Future

Over time, there are more higher-level-of-evidence studies being completed in the area of pediatric robotic surgery (4). The largest randomized trial completed, to date, showed no benefit for robotic surgery for radical prostatectomy (15). However, there is a need for additional randomized trials comparing robot-assisted surgery to open or traditional laparoscopic surgery. It has been suggested that we are reaching a “tipping point” as indicated by the larger number of publications and reported case volumes (24, 25). In a survey of 117 pediatric surgeons, the majority felt that robotic surgery has a future role, although over 80% of respondents had no personal experience with robotic surgery (26). Robotic surgery has established itself in the adult population but due to technical and financial limitations specific to pediatrics, it may be some time before we see the same popularity in pediatric surgery.

## Conflict of Interest Statement

The authors declare that the research was conducted in the absence of any commercial or financial relationships that could be construed as a potential conflict of interest.
